# The Effect of COVID-19 on Health Care Utilization Among Children with Medical Complexity: Retrospective Chart Review Study

**DOI:** 10.2196/65751

**Published:** 2025-02-28

**Authors:** Onur Asan, Ilse Super, Stephen Percy, Katharine N Clouser

**Affiliations:** 1 Department of Systems and Enterprises, Stevens Institute of Technology, 1 Castle Terrace Dr, Hoboken, NJ, 07030, United States, 1 201-216-5514; 2 Hackensack Meridian School of Medicine, Hackensack University Medical Center, Hackensack, NJ, United States

**Keywords:** children with medical complexity, pediatric, children, health care utilization, telemedicine, telehealth, virtual care, virtual health, COVID-19, SARS-COV-2, coronavirus, respiratory, infectious, pulmonary, pandemic, chart review, chart review study, retrospective chart review, retrospective chart review study

## Abstract

This study examines the trends, patterns, and potential health disparities in health care utilization among children with medical complexity, before and during COVID pandemic through a retrospective chart review. Our findings show significant differences in the average number of visits per patient over the years and support the adoption of telehealth consultations, while highlighting concerns about demographic disparities.

## Introduction

Children with medical complexity are defined as children and youth with chronic and severe health conditions and substantial functional limitations that require specialized medical services, frequent hospitalizations, and coordinated care from various health care providers [1,2].

Telehealth was one of the efficient and creative solutions for care for children with medical complexity during the pandemic [3]. These children face numerous barriers to health care (eg, multiple visits per day, limited access and availability of clinicians, high cost of transportation, day-to-day life disruptions) [4]. Telehealth visits or remote virtual consultations offered numerous benefits at the onset of the COVID-19 pandemic, such as limited exposure, fewer transfers, and minimum travel for several types of patients and specialists, while also increasing the availability of consultations [5]. This alternative to in-person visits has proven to be effective in resource-limited countries with geographical barriers and has reduced the burden of negative consequences for chronically ill children [6]. After its rapid implementation during the pandemic, the attention has shifted towards equity and safety for children with medical complexity postpandemic, while focusing on clinical model refinement and addressing potential health disparities [7,8]. We explored health care utilization patterns among children with medical complexity over 3 years, before and after the pandemic.

## Methods

### Study Design and Participants

We performed a retrospective chart review study using data from a large health care setting on the East Coast between January 1, 2019, and December 31, 2021. Patients that were included in the study (N=435) were children with medical complexity (<22 years of age), diagnosed with ≥3 chronic conditions [9]. All patient mortality cases (n=5) during the selected period were excluded due to incomplete data for comparison.

### Ethical Considerations

This study used deidentified chart data retrieved from electronic health records using *ICD-10* codes to identify the population. The data were fully anonymized before analysis, and no personal or identifiable information was accessed. The study was conducted in compliance with all relevant data protection regulations, including the HIPPA (Health Insurance Portability and Accountability Act). Institutional Review Board approval was obtained (IRB ID: Pro2023-0385) from Hackensack Meridian Health.

### Data Processing and Analysis

A demographic summary and descriptive statistics were computed to analyze the distribution of demographic variables, years, and health care visit types, including counts, means, and standard deviations. To evaluate associations between these variables, the Pearson *χ*^2^ tests of independence were conducted. For variables showing significant associations (*P*<.05), we reported the effect size using Cramer’s V [5] to determine the strength of the relationship. Additionally, posthoc pairwise comparisons were performed using *χ*^2^ tests with adjusted significance levels to identify specific category pairs contributing to the observed differences. All analyses were performed using Microsoft Excel and SPSS software (version 29; IBM Corp) to ensure accuracy and consistency.

## Results

### Demographic Summary

Table 1 presents the demographic summary of the patients included in the study.

**Table 1.** Demographic summary of patients.

Variables	Patients, n (%) (N=435)
Age	
Infants (1-2)	55 (12.6)
Children (3-11)	231 (53.1)
Adolescents (12-18)	104 (23.9)
Young Adults (18-21)	45 (10.3)
Race/ethnicity	
Asian	65 (14.9)
Black	65 (14.9)
Hispanic	82 (18.9)
NonHispanic White	85 (29.5)
Other	67 (15.4)
Unknown	71 (16.3)
Insurance method	
Managed Care	302 (69.4)
Private	99 (22.8)
Other	34 (7.8)

### Statistical Analysis

Table 2 provides descriptive statistics for the entire dataset. The data reveal that the highest total average was 16.74 appointments per patient before the pandemic in 2019. In 2020, with the onset of COVID-19, telehealth became a viable means of communicating with patients, while other types of visits and inpatient admissions decreased, resulting in a total average of 10.34 appointments per patient. Although mean of all visit types, including inpatient admissions increased in 2021, they did not return to the 2019 levels, leading to an overall average of 13.22 appointments per patient.

**Table 2.** Data description and findings. This table describes the mean and standard deviations of the number of visits for each visit type (telehealth, outpatient, emergency department) and admissions per patient.

Demographic variables	Visit types															
	Telehealth visits, mean (SD)				Outpatient visits, mean (SD)				Emergency department visits, mean (SD)				Inpatient admissions, mean (SD)			
	2019	2020	2021	*P* value	2019	2020	2021	*P* value	2019	2020	2021	*P* value	2019	2020	2021	*P* value
Overall^a^	0 (0)	2.84 (2.02)	2.01 (1.41)	<.001	6.01 (1.98)	3.50 (1.09)	5.33 (2.31)	<.001	7.31 (2.28)	2.44 (1.70)	3.40 (2.21)	<.001	3.42 (2.30)	1.56 (1.11)	2.48 (1.69)	<.001
Age^b^				.78				.15				.45				.86
Young adults	0 (0)	2.44 (2.23)	2.22 (1.41)		5.91 (1.78)	3.96 (1.02)	4.91 (2.33)		7.07 (2.32)	2.69 (1.49)	4.07 (2.04)		3.71 (2.14)	1.60 (1.16)	2.27 (1.47)	
Children	0 (0)	2.99 (1.94)	1.99 (1.39)		5.81 (2.06)	3.50 (1.08)	5.37 (2.27)		7.32 (2.32)	2.45 (1.72)	3.42 (2.19)		3.31 (2.35)	1.61 (1.09)	2.45 (1.73)	
Adolescents	0 (0)	3.09 (2.07)	1.99 (1.43)		6.17 (1.94)	3.46 (1.10)	5.35 (2.37)		7.24 (2.25)	2.49 (1.67)	3.21 (2.23)		3.61 (2.35)	1.51 (1.12)	2.52 (1.70)	
Infants	0 (0)	2.05 (1.87)	1.98 (1.46)		6.62 (1.79)	3.24 (1.09)	5.45 (2.36)		7.62 (2.16)	2.11 (1.80)	3.11 (2.33)		3.31 (2.13)	1.40 (1.13)	2.73 (1.72)	
Race/ethnicity^b^				.30				.04				.86				.85
Asian	0 (0)	2.80 (2.03)	2.26 (1.47)		5.49 (1.80)	3.35 (1.02)	5.51 (2.37)		7.35 (2.34)	2.46 (1.71)	3.52 (2.03)		3.51 (2.24)	1.71 (1.10)	2.71 (1.68)	
Black	0 (0)	2.91 (2.26)	2.09 (1.51)		6.46 (1.76)	3.51 (1.09)	5.22 (2.23)		7.22 (2.26)	2.51 (1.69)	3.86 (2.24)		3.37 (2.22)	1.54 (1.13)	2.31 (1.66)	
Hispanic	0 (0)	2.52 (1.95)	1.79 (1.39)		5.98 (1.81)	3.45 (1.11)	4.96 (2.19)		7.63 (2.27)	2.66 (1.76)	3.24 (2.30)		3.72 (2.22)	1.61 (1.04)	2.44 (1.74)	
NonHispanic White	0 (0)	2.65 (1.93)	2.02 (1.37)		6.49 (2.07)	3.71 (1.14)	5.38 (2.33)		7.31 (2.08)	2.13 (1.54)	3.40 (2.24)		3.41 (2.21)	1.59 (1.14)	2.35 (1.82)	
Other	0 (0)	3.30 (2.04)	1.87 (1.28)		5.60 (2.11)	3.66 (1.05)	5.30 (2.41)		7.10 (2.57)	2.57 (1.79)	3.36 (2.31)		3.06 (2.42)	1.57 (1.05)	2.66 (1.67)	
Unknown	0 (0)	2.97 (1.93)	2.08 (1.44)		5.93 (2.13)	3.31 (1.08)	5.65 (2.37)		7.18 (2.26)	2.35 (1.71)	3.08 (2.09)		3.39 (2.36)	1.32 (1.18)	2.46 (1.56)	
Insurance methods^b^				.86				.44				.43				.17
Managed Care	0 (0)	2.81 (2.02)	1.99 (1.42)		6.06 (1.98)	3.52 (1.07)	5.23 (2.34)		7.32 (2.27)	2.44 (1.66)	3.42 (2.19)		3.39 (2.30)	1.56 (1.11)	2.43 (1.68)	
Private	0 (0)	2.78 (1.99)	2.04 (1.39)		5.92 (1.98)	3.59 (1.11)	5.63 (2.34)		7.28 (2.36)	2.38 (1.77)	3.35 (2.20)		3.40 (2.36)	1.49 (1.12)	2.55 (1.74)	
Other	0 (0)	3.26 (2.09)	2.09 (1.38)		5.85 (2.06)	3.15 (1.16)	5.29 (1.93)		7.29 (2.21)	2.62 (1.86)	3.32 (2.50)		3.74 (2.09)	1.74 (1.08)	2.76 (1.69)	

a Overall averages for each year with a *P* value for difference between the years

b Averages per year for each demographic group with a *P* value for the difference between demographic categories*.*

A *χ*^2^ test of independence revealed significant differences between the years for telehealth visits (*P*<.001, Cramer’s V=0.612), outpatient visits (*P*<.001, Cramer’s V=0.396), emergency department visits (*P*<.001, Cramer’s V=0.557), and inpatient admissions (*P*<.001, Cramer’s V=0.405).

Among the demographic variables, a significant association was found between race or ethnicity and the number of outpatient visits for three years (*P*=.04, Cramer’s V=0.008). Pairwise comparison for outpatient visits revealed three significant relationships between Asian (*P*=.02), Hispanic (*P*=.004), and other patients (*P*=.02) compared to nonHispanic White patients.

## Discussion

Telehealth visits were introduced in 2020, and resulted in a notable decrease in other visit types and inpatient admissions, as reported in other studies [4]. Although 2021 saw an increase in these visit types compared to 2020, they did not return to prepandemic levels observed in 2019. This could be attributed to catch-up visits and admissions that were postponed due to safety reasons during the pandemic [10]. In addition, while many visits can be conducted via telehealth systems, some health care procedures require in-person visits; the safety of these in-person visits improved in 2021.

The reduction in in-person visits after introducing telehealth implementation aligns with another study showing lower hospitalization rates 3 months post discharge when telehealth was used for follow up [11]. Our study also found a reduction in emergency department visits after telehealth introduction, as shown in the literature [8]. Increasing the ease at which a family can access health care via telehealth can avoid some of the in-person struggles faced by families and improve satisfaction with health care interactions.

Our findings show that Asian, Hispanic, and other racial and ethnic groups, have significantly fewer outpatient visits, on average, than nonHispanic White patients. These findings partially align with recent research among Medicaid-insured children with medical complexity, which found that Black nonHispanic and Hispanic children had lower outpatient visit rates than nonHispanic White children [12]. Several reasons may contribute to lower out-patient visits among racial and ethnic minorities, including differential access to care [12], geographical dispersion, a reduced likelihood of receiving specialty referrals from primary care providers, and distrust in the health care system [13]. Significant differences in the number of visits could lead to health disparities, potentially disadvantaging minority groups, and should, therefore, be monitored and addressed.

The COVID-19 pandemic has shown the feasibility of telehealth visits. Although we found disparities based on race and ethnicity in outpatient visits, our findings support equity through telehealth visits for patients and reduced health care utilization. There was no increase in the need for inpatient services when using telehealth visits, which supports the idea that telehealth can be an effective solution for patients. Families can avoid transportation challenges and coordination issues when using telehealth visits.

Our study focuses on changes in health care utilization among children with medical complexity with 3 or more chronic conditions, providing valuable insights into trends within this population. However, we did not account for specific illnesses, clinical outcomes, or the quality of telehealth visits. Additionally, we did not consider environmental factors such as air pollution or pollen exposure, which could significantly influence utilization patterns among children with respiratory or allergic conditions [14]. To deepen our understanding, future research should include control groups or broader pediatric populations to contextualize these observed trends and address potential confounding factors. Additionally, qualitative analyses are needed to explore the reasons behind differences in utilization and disparities, offering a more comprehensive perspective on the challenges faced by this population.

In conclusion, our findings emphasize the need for continued adaptation and support for telehealth services among children, while monitoring demographic disparities in health care access. The introduction of telehealth at the onset of the pandemic coincided with significant decreases in in-person visits and inpatient admissions. Although health care utilization rebound in 2021, it remained below prepandemic levels, suggesting an ongoing adjustment in health care practices. Further, our findings indicate that racial and ethnic disparities persist in outpatient visit patterns. Continued support for telehealth implementation, coupled with targeted efforts to address disparities, are crucial for equitable health care access for all children with medical complexity.

## Abbreviations

HIPPA: Health Insurance Portability and Accountability Act
